# The Public Services

**Published:** 1909-09-04

**Authors:** 


### The Public Services.

### THE NAVY MEDICAL DEPARTMENT.

This Service is only open to the registered graduate of European birth between 21 and 28 years of age. He must pass an entrance examination and gain at least one-third of the possible marks, and he must satisfy the physical requirements of the Medical Board.

The examination will consist of two parts, as follows :—(i.) compulsory subjects, and (ii.) voluntary subjects. The former are (*a*) medicine, materia medica, therapeutics, and general hygiene; (*b*) surgery and surgical anatomy; (*c*) pathology. The attention of candidates is especially drawn to the importance of the section of Operative Surgery. No candidate will be considered eligible who has not obtained at least one third of the maximum marks in each of the above compulsory subjects. The voluntary subjects are French and German. The knowledge of modern languages being considered of great importance by the authorities, all intending competitors are urged to qualify in French and German.

Successful candidates immediately after passing the examination will receive commissions as surgeons in the Royal Navy, and undergo a course of practical instruction in naval hygiene, the diseases of warm climates, bacteriology, surgery of gunshot wounds, etc., radiography, and the photography in connection with it, at Haslar Hospital.

Every medical officer will be required to undergo a post-graduate course of three months' duration at a metropolitan hospital once in every eight years (should the exigencies of the service permit), and this as far as possible during his surgeon's, staff surgeon's, and fleet surgeon's period of service. While carrying out this course the medical officer will be borne on a ship's books for full pay, and will be granted lodging and provision allowances, and travelling expenses as for service under the regulations to and from his home or port; the fees for each course (not exceeding £25) will be paid by the Admiralty on the production of vouchers at the end of the course. The medical officer will be required to produce separate certificates of efficient attendance in the following: I. The medical and surgical practice of the hospital; II. A course of operative surgery on the dead body; III. A course of bacteriology; IV. A course of ophthalmic surgery, particular attention being paid to the diagnosis of errors of refraction; V. A practical course of skiagraphy.

### THE ROYAL ARMY MEDICAL CORPS.

A candidate for a commission in the Royal Army Medical Corps must be 21 years and not over 28 years of age, and must possess a registered qualification to practise. He must pass an entrance examination, and, having gained a place and having satisfied the Medical Board of his physical fitness, he must undergo two months' instruction in hygiene and bacteriology, and afterwards proceed to Aldershot for instruction in the technical duties of the corps.

The entrance examination is held twice a year in London, usually in January and July, and an entrance fee of £1 is charged for admission to it. It lasts about four days. The examination is of a clinical and practical character, partly written and partly oral. It consists of examination and report upon a medical case and commentary upon a case in medicine; clinical medical cases and *viva voce* on medical pathology; examination and report upon a surgical case and commentary upon a case in surgery; clinical surgery and pathology (including diseases of the eye), and operative surgery, bandaging, surgical instruments and appliances.

After the lieutenant on probation has passed the examinations in the subjects taught, and has satisfied the director-general that he possesses the necessary skill, knowledge, and character for permanent appointment to the Royal Army Medical Corps, his commission as lieutenant will be confirmed. A lieutenant on probation who, at the time of passing the examination for admission to the Royal Army Medical Corps, holds, or is about to hold, a resident appointment in a recognised civil hospital, may be seconded for the period, not exceeding one year, during which he holds the appointment. While seconded he shall not receive pay from Army funds, but his service shall reckon towards promotion, increase of pay, gratuity, and pension.

### THE INDIAN MEDICAL SERVICE.

Candidates must be natural-born subjects of His Majesty, of European or East Indian descent, between 21 and 28 years of age at the date of the examination, of sound bodily health, and in the opinion of the Secretary of State for India in Council in all respects suitable to hold commissions in the Indian Medical Service. They may be married or unmarried. They must possess, under the Medical Acts in force at the time of their appointment, a registrable qualification to practise both medicine and surgery in Great Britain and Ireland.

The physical fitness of each candidate will be determined by a Board of Medical Officers, who are required to certify that his vision is sufficiently good to enable him to pass the tests laid down by the Regulations. The candidate will be examined by the Examining Board in the following subjects: (1) medicine, including therapeutics; (2) surgery, including diseases of the eye; (3) applied anatomy and physiology; (4) pathology and bacteriology; (5) midwifery and diseases of women and children; (6) materia medica, pharmacology, and toxicology. The examination in medicine and surgery will be in part practical, and will include operations on the dead body, the application of surgical apparatus, and the examination of medical and surgical patients at the bedside. No syllabus is issued in the subjects of pharmacology and toxicology, but the examination will be conducted so as to test the general knowledge of the candidate in these subjects. No candidate shall be considered eligible who shall not have obtained at least one-third of the marks obtainable in each of the above subjects and one-half of the aggregate marks for all the subjects.

After passing examination, the successful candidates will be required to attend one entire course of practical instruction at the Army Medical School and elsewhere, as may be decided, in: (1) hygiene; (2) military and tropical medicine; (3) military surgery; (4) pathology of diseases and injuries incidental to military and tropical service. This course will be of not less than four months' duration, and at its conclusion candidates will be required to pass an examination in the subjects taught therein.

### THE COLONIAL MEDICAL SERVICE.

In the following countries there are medical departments regulated from the Colonial Office :—Jamaica, Trinidad, Tobago, British Guiana, Windward and Leeward Islands, British Honduras, Fiji, Ceylon, Straits Settlements, Federated Malay Straits, Hong-Kong, British East Africa, Uganda, Mauritius, Seychelles, Gibraltar, St. Helena, Falkland Islands, Cyprus, Gambia, Sierra Leone, the Gold Coast, Northern and Southern Nigeria. The last four of the countries named have been formed into the West African Medical Staff.

